# Effects of mindfulness-based cognitive therapy on older adults with sleep disorders: a systematic review and meta-analysis

**DOI:** 10.3389/fpubh.2023.1242868

**Published:** 2023-12-18

**Authors:** Ana María González-Martín, Agustín Aibar-Almazán, Yulieth Rivas-Campo, Alejandro Marín-Gutiérrez, Yolanda Castellote-Caballero

**Affiliations:** ^1^Department of Education and Psychology, Faculty of Social Sciences, University of Atlántico Medio, Las Palmas de Gran Canaria, Spain; ^2^Department of Psychology, Centro de Educación Superior de Enseñanza e Investigación Educativa, Madrid, Spain; ^3^Department of Health Sciences, Faculty of Health Sciences, University of Jaén, Jaén, Spain; ^4^Faculty of Human and Social Sciences, University of San Buenaventura-Cali, Santiago de Cali, Colombia

**Keywords:** mindfulness, sleep disorders, older adults, systematic review, meta-analysis

## Abstract

**Objective:**

This systematic review and meta-analysis was conducted to analyze the effectiveness of a mindfulness-based program on sleep quality in healthy non-institutionalized older people.

**Methods:**

This study was conducted following the PRISMA (Preferred Reporting Items For Systematic Reviews And Meta-Analyses) guidelines. The search was conducted during May and June 2023 using four databases: Pubmed, Scopus, Web of Science, and CINAHL. Different keywords combined with Boolean operators were used. Only 10 articles of the initial 177 were included. In the study, the standardized mean difference (SMD) was used along with a 95% confidence interval to measure the effect. Heterogeneity among the studies, assessed using Cochran's *Q*-test and the *I*^2^ statistic was found to be low, leading to the use of a fixed-effects model in the analysis. The effect size was expressed as Hedge'g. Furthermore, a subgroup analysis was conducted, taking into account the various tools used to assess sleep conditions.

**Results:**

Mindfulness was found to reduce poor sleep quality in people with both long-term and short-term sleep disorders. Weighting effect model Hedge'g = −0.344 with a 95% confidence interval ranging from −0.425 to −0.263. In all cases, statistically significant results were observed, as well as moderate and negative effect sizes according to the Hedge's g index: −0.326 for Insomnia Severity Index (ISI), −0.343 for Pittsburgh Sleep Quality Index (PSQI), and −0.28 for Sleep Onset Latency (SOL).

**Conclusion:**

This systematic review and meta-analysis found that mindfulness can be used to remedy poor sleep quality in older people, so it could be a viable treatment option for insomnia or other problems related to poor sleep quality in this population.

## 1 Introduction

Sleep disorders are a widespread public health problem affecting between 20% and 30% of adults (1). They have numerous adverse consequences on individual quality of life and place a significant economic burden on society. Poor sleep quality is more common among adults over the age of 60 because this age group has a higher prevalence of sleep problems than younger age groups when measured through biological assessments or self-reports (2). This poor sleep quality is related to multiple factors such as nighttime worry and chronic stress, which subsequently affect health outcomes, including increased morbidity and mortality, as well as reduced quality of life (3–5). In numerous studies, about half of older adults have reported sleep problems, including difficulty falling asleep, sleep disruption, and general dissatisfaction with sleep quality and quantity (6, 7).

Generally, specialists argue that the reduction in sleep quality is not a direct consequence of normal aging but a consequence of different aging-related factors or processes that lead to difficulty sleeping (4, 8). Currently, insomnia treatment procedures incorporate pharmacological and behavioral treatments (9), but each has a series of limitations that impair their impact. Although hypnotic medications can decrease sleep latency and increase total nighttime sleep (10, 11), significant concerns have been raised related to drug tolerance and dependence, as well as potential side effects such as acute memory disturbances, impaired balance and gait, and residual daytime sleepiness. Therefore, most patients prefer non-pharmacological strategies or treatments (12, 13), of which psychotherapy is one option to consider, but this requires a time contribution from health professionals (14).

Psychobehavioral therapies are also recognized as non-pharmacological treatments for sleep disorders (9). A universally known behavioral program is sleep hygiene education, characterized primarily by changing the environmental factors and everyday behaviors that negatively affect sleep deprivation (15). Within standard clinical treatments, cognitive behavioral therapy focuses on modulating sleep needs and modifying attitudes, expectations, and beliefs about sleep (16). These non-pharmacological treatments have several advantages over pharmacotherapy due to their effectiveness in improving both long- and short-term sleep while showing no serious contraindications (17). However, interventions like cognitive behavioral therapy are intensive, aimed at patients with sleep problems, and require the intervention of therapists with high levels of knowledge (18).

The limitations of recent treatments for sleep problems highlight the need for affordable treatments for sleep improvement among older adults with moderate sleep disorders (19). Of these treatments, mindfulness-based interventions (MBI) have the potential capacity to meet these needs as they are characterized as evidence-based programs for stress-related ailments (20) that train the person in the systematic practice of paying attention to experiences, emotions, and thoughts (21). Evidence from previous studies (22, 23) indicates preliminary but mixed support for the use of MBIs for sleep disorders in adults, and a systematic review of the effectiveness of MBIs for sleep problems highlighted a range of gaps in the existing research (24). Sleep has been mainly evaluated as a secondary outcome of a primary pathological state that can disrupt sleep (25, 26), so the findings may be confused due to changes in the primary ailment. Overall, the existing MBI studies need to be updated to determine the optimal intervention protocols in non-institutionalized older people.

Therefore, the objective of this systematic review and meta-analysis was to analyze the effectiveness of a mindfulness-based program on sleep quality in healthy non-institutionalized older people.

## 2 Materials and methods

A systematic review with meta-analysis was conducted to determine the effects of mindfulness-based cognitive therapy in older adults with sleep disorders. The review was conducted following the PRISMA (Preferred Reporting Items For Systematic Reviews And Meta-Analyses) 2020 guidelines and the Cochrane Handbook for the Elaboration of Systematic Reviews of Interventions, as proposed by Higgins et al. (27). The review protocol was pre-specified in PROSPERO and registered under the code CRD42023424438.

### 2.1 Sources of information

A bibliographic search was carried out using the Pubmed, Scopus, Web of Science, and CINAHL databases during May and June 2023.

### 2.2 Search strategy

(“Mindfulness-based cognitive therapy” OR “MBCT” OR “mindfulness based therapy” OR “Mindfulness based intervention” OR “MINDFULNESS SLEEP THERAPY”) AND (“Older adults” OR “ELDERLY” OR “Aged”) AND (“sleep quality” OR “Sleep disorders” OR “SLEEP HYGIENE”).

### 2.3 Inclusion criteria

Included articles had to meet the following criteria: (i) Randomized controlled clinical trials (RCTs) using objective measures to assess insomnia, sleep quality, or hypersomnia in older adults; (ii) Types of intervention: mindfulness-based cognitive therapy or mindfulness-based therapy as a treatment; (iii) Languages of the study: English or Spanish; (iv) Published from January 2010 to June 2023.

Additionally, the researchers imposed a rigorous time frame for the inclusion of publications. This time interval encompassed a 12-year period, ranging from January 2010 to June 2023. The rationale behind this choice was based on the belief that evaluating the effects of mindfulness application within their field of study required a substantial amount of time to gain wide recognition and adoption within the academic and scientific community. Establishing this 12-year window enabled them to comprehensively capture the most up-to-date literature related to their research topic while ensuring that the selected publications were aligned with the most recent developments in the field.

### 2.4 Exclusion criteria

They discarded studies that did not meet an acceptable level of internal validity (i.e., those with a score below six on the PEDro scale) and external validity. Additionally, publications such as books or articles, meta-analyses, reviews, systematic reviews, protocols, clinical trial registries, and articles that had not undergone peer review were excluded from consideration. Furthermore, studies that focused on ethnic minorities, individuals with limited mobility, acute infections, and hormonal disorders were also excluded. These exclusion criteria were implemented to ensure the integrity and validity of the information used in their work, with a priority placed on the inclusion of research supported by rigorous peer review and a solid scientific foundation.

### 2.5 Study selection process

The search results were processed using the Rayyan QCRI (https://rayyan.qcri.org/welcome) application. Duplications were eliminated. Two authors reviewed the titles and abstracts of the articles and excluded those that failed to meet the inclusion criteria. This task was performed blindly. Two authors independently and blindly verified compliance with the inclusion criteria and read the full articles. Differences arising during this process were resolved by reaching a consensus with a third author.

### 2.6 Data extraction

The main variables used in the review focus on the measurement of outcomes of sleep disorders. Each article was classified according to the type of disorder evaluated, year of publication, country, author/s, characteristics of the participants (age, inclusion and exclusion criteria, sample size, and group distribution), details of the intervention (duration, frequency), types of variables, tests used, and follow-up time.

### 2.7 Evaluation of methodological quality

The methodological quality of the selected articles was evaluated using the Physiotherapy Evidence Database (PEDro) scale, one of the most common scales for assessing this feature. This tool is specially designed to assess the methodological quality of randomized controlled trials; it consists of 11 items that address various aspects of the study methodology, such as random allocation, allocation concealment, blinding of participants and assessors, patient follow-up, among others. Each item is scored dichotomously (1 if the criterion is met, 0 if it is not), and the total score is used to determine the methodological quality of the study (28). Scores were sought on the PEDro website whenever they were available, the maximum being 10 points (29). When these were unavailable, two authors assessed the methodological quality of the articles, with a third author resolving any discrepancies that arose.

### 2.8 Analytical decisions for meta-analysis

Statistical estimators were employed to synthesize the findings, specifically the mean difference (SMD) and a 95% confidence interval (CI), or the risk ratio (RR) along with a 95% CI, as applicable. Heterogeneity was assessed through Cochran's *Q*-test, indicating significant heterogeneity when present, and the *I*^2^ statistic, which quantified the proportion of total variability.

Subsequently, a subgroup analysis was conducted, taking into consideration the specific tool used to evaluate sleep conditions. This analysis aimed to explore whether variations in results were associated with these distinct assessment methods. Furthermore, to evaluate potential publication bias, a funnel plot was employed.

In the results analysis phase, a sensitivity analysis was undertaken to examine the impact of individual studies or key variables on the overall outcomes. This analysis was performed through subgroup analyses that considered the sleep condition assessment tool, investigating whether the results exhibited variations based on these specific criteria. Additionally, publication bias was assessed using a funnel plot.

The research employed Comprehensive Meta-Analysis (CMA) software version 3.0, developed by Biostat, Inc. in the United States. This software is widely acknowledged and utilized for conducting meta-analyses across various research disciplines.

## 3 Results

### 3.1 Selection of the studies

**Figure 1:**
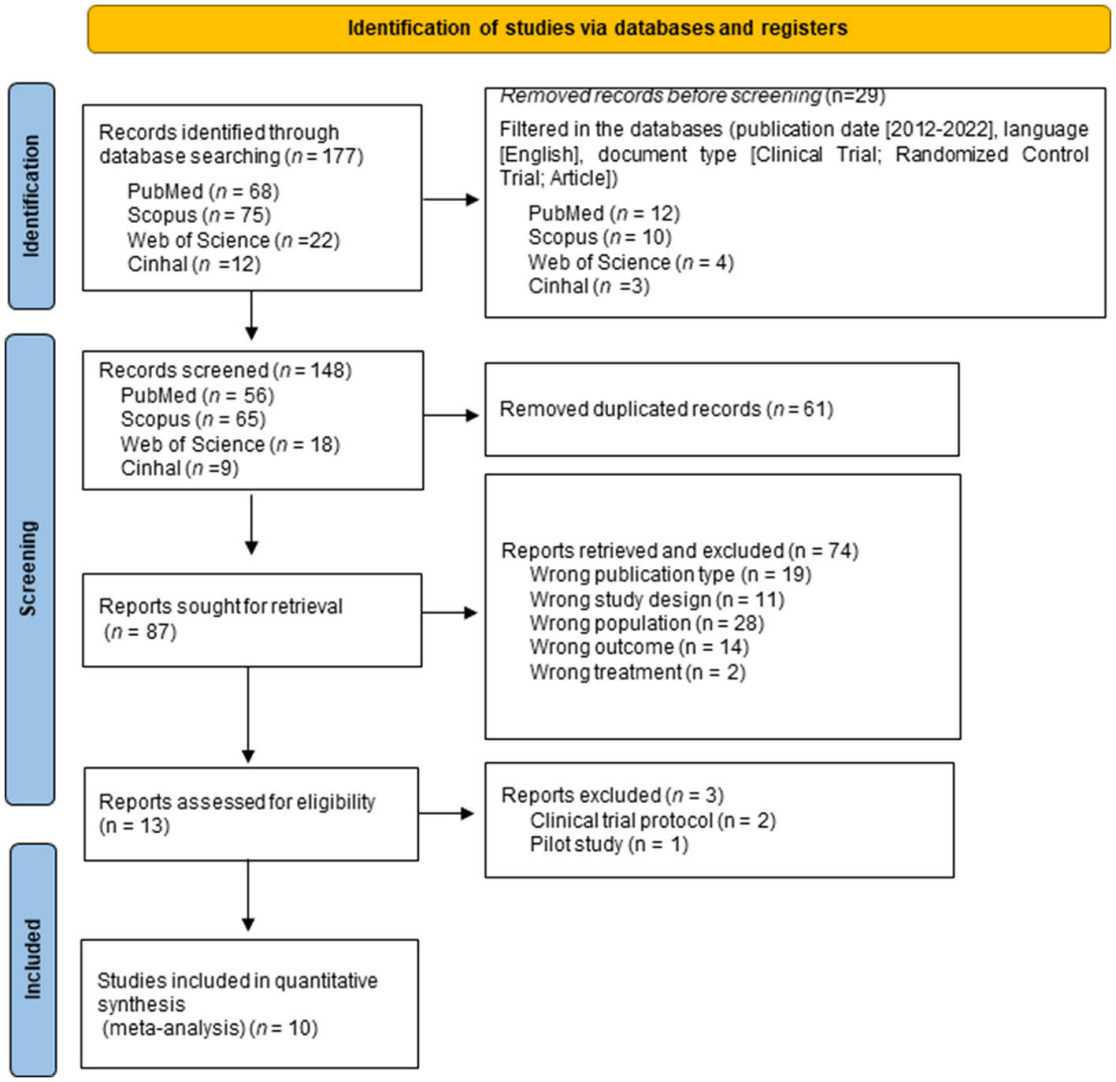
Flow diagram of the study selection process.

Complete searches were performed in the different databases, resulting in a total of 177 articles; the number was reduced to 148 when applying the automation filters. Subsequently, 61 duplicate articles were eliminated, leaving a total of 87 articles to be evaluated. Once the evaluation was completed, the articles were analyzed for eligibility, with only 10 articles (30–39) meeting the inclusion criteria (Figure 1).

### 3.2 Methodological quality

The PEDro scale was used to assess methodological quality. The scores for two studies (32, 33) were obtained from the PEDro website, while the others (30, 31, 34–39) were evaluated manually. Of all the articles included, seven were classified as having “good” (30, 32–35, 37, 39) methodological quality, while three obtained an “excellent” (31, 36, 38) classification.

### 3.3 Characteristics of the studies

The articles selected in this systematic review corresponded to randomized controlled clinical trials published in English (30–39) from 2012 to 2022, as well as in 2012 (33), 2014 (38), 2015 (36, 37), 2017 (32), 2018 (35), and 2022 (30, 31, 34, 39). They originated from different countries: Spain (30), Singapore (31, 33), China (32, 34, 36, 39), and the USA (35, 37, 38). Four articles focused primarily on insomnia (30, 32, 33, 38) while the other six addressed overall sleep disorders (31, 34–37, 39). A total of 1,218 people participated in the selected studies, of which 605 belonged to experimental groups. The sample sizes used in the 10 articles included in this systematic review ranged from 47 (30) to 209 individuals (34).

In terms of duration, the mindfulness-based cognitive therapy interventions generally lasted 8 weeks (30–36, 39). Two investigations were extended with follow-up to 2 months (32, 35), one was extended to 4 months (34), and the longest had a follow-up at 16 months (38). Table 1 contains the full details of the articles selected in this review.

**Table 1:** Characteristics of the included studies.

**Country**	**References**	**Population**	**Sample CG/IG**	**Control group**	**Intervention group**				**Results**	**Values post treatment**
					**Age**	**Intervention type**	**Measuring instrument**	**Assessments**		
Spain	Camino et al. (30)	Older adults with subclinical and moderate insomnia	CG = 24 • EG = 23	Eight-week film-forum activity (coinciding with the intervention weeks) on active aging. After the activity ended, participants were also invited to a group session to conduct the post-intervention assessment.	72.9 (6.6)	Treatment that combines mindfulness and cognitive therapy is effective for treating insomnia in older adults.	Pittsburgh Sleep Quality Index	T0 = Baseline • T1 = 8 weeks	Results were obtained on both scales, with a reduction in insomnia symptoms in the subclinical and moderate intervention groups. The intervention group obtained significantly decreasing effects on subjective sleep quality, *F*_(1, 45)_ = 15.25, *p* = 0.027, η^2^ = 0.105. The control group showed no significant effects on subjective sleep quality, *F*_(1, 45)_ = 0.66, *p* = 0.419, η^2^ = 0.015	*p* < 0.001
							Insomnia Severity Index (ISI)		ISI: simple effects tests showed a significantly decreasing effect in the intervention group, *F*_(1, 45)_ = 10.41, *p* = 0.002, η^2^ = 0.188, whereas the effect was not significant in the control group, *F*_(1, 45)_ = 0.95, *p* = 0.334, η^2^ = 0.021.	*p* = 0.01
Singapore	Shaif et al. (31)	Older adults with sleep disorders	CG = 55 • EG = 58	Eight weekly sessions of 2 h. Each session introduced a concept related to sleep and sleep hygiene. Participants received a manual describing the concept and how they intended to apply it in their daily lives.		Eight 2-h sessions covering various mindfulness techniques (e.g., mindfulness of breathing, body and movement, senses and informal practice, and empathy and compassion) pertaining to people with sleep problems and insomnia. Participants received brochures with the information covered during the talks and discussions.	Sleep discrepancies at sleep onset latency (SOL) and awakening after sleep onset (WASO). The SOL discrepancy was measured by polysomnography/ ISI.	T0 = Baseline • T1 = 8 weeks	The sleep onset latency discrepancy, measured by polysomnography and actigraphy, decreased significantly after MBTI and SHEEP interventions. In contrast, no significant changes in wakefulness occurred after the discrepancy in sleep onset in either group.	SOL Dif = 18.24 *p* = 0.036/ISI Mean difference: 5.02 *t*-value: 8.499 • *p* < 0.001 Cohen's *d* = 1.135
China	Wong et al. (32)	Older adults with insomnia	CG = 95 • EG = 101	Eight weeks of sleep psycho-education with exercise control (PEEC)	58 (9.1)	Eight weeks of mindfulness-based cognitive therapy for insomnia (MBCT-I)	Insomnia Severity Index (ISI), Insomnia Severity Index; FFMQ, the Five Facet Mindfulness Questionnaire; SOL, sleep onset latency; WASO, wake time after sleep onset; TST, total sleep time	T0 = baseline T1 = 2 months (post-intervention) • T2 = 5 months (3-month follow-up), and • T3 = 8 months (6-month follow-up)	The ISI score significantly decreased in the MBCT-I group compared to the PEEC group at 2 months (i.e., post-intervention) (*p* = 0.023, effect size [95% CI] −0.360 [−0.675, −0.046]) but not at 5 or 8 months.	ISI t0 = 18.2 (3.8) t1=-4.0 (3.7) t3= −5.2 (4.1)
Singapore	Perini et al. (33)	Older adults with insomnia	CG = 62 • EG = 65	Sleep hygiene, education, and exercise (SHEEP) program on sleep biology and self-monitoring sleep behavior showed changes in habits and environment that could improve sleep quality.	60.9 (6.4)	Eight weekly sessions of 2 h each of MBTI mindfulness-based therapy for insomnia.	Insomnia Severity Index (ISI)	T0 = Baseline • T1 = 4 weeks • T2 = 8 weeks	Significant interaction of time × group [*F*_(125.1)_ = 6.89, *p* = 0.010], with MBTI showing a significantly greater reduction in insomnia severity than SHEEP. Estimation analysis confirmed that both groups improved from baseline [MBTI: *d* = −1.27, 95% confidence interval (CI) −1.61 to −0.89; SHEEP: *d* = −0.69, 95% CI −0.96 to −0.43].	IG T0 = 14.89 (3.89) • T2 = 9.95 (3.88) • CG T0 = 14.21 (4.13) • T2 = 11.23 (4.54) *p* = 0.34
							Pittsburgh Sleep Quality Index (PSQI)	T0 = Baseline •T1 = 4 weeks • T2 = 8 weeks	Both groups reported better sleep quality scores over time without significant time interactions × group on the PSQI.	T0 = 10.98 (3.10) T1 = 9.14 (2.74) T2 = 7.34 (3.01)
China	Lee et al. (34)	Older adults	CG = 104 • EG = 105	Control group (received the mindfulness program 8 weeks later; i.e., immediately after participants in the intervention group received the mindfulness program).	CG = 71.8 (8.2) • EG = 71.3 (7.0)	Eight-week modified mindfulness-based stress reduction (mMBSR). Participants were asked to do mindful home practices six times per week.	Pittsburgh Sleep Quality Index (PSQI)	T0 = Baseline T1 = 5 months • T2 = 4 months	There was a change in the PSQI at 2 months: 8.3–6.7, *p* < 0.001. The change was maintained at 4 months.	EG) T0 = 8.3 (4.9) T1 = 6.7 (4.5) < 0.001 T2 = 6.6 (4.9) *p* < 0.001 CG T0 = 6.5 (4.3) T1 = 5.5 (4.2) *p* = 0.002
USA	Gallegos et al. (35)	Older adults with sleep disorders	CG = 100 • EG = 100	Wait-list control group (WLC)	CG = 73 (6.59) • EG = 72 (6.82)	Eight-week mindfulness-based stress reduction (MBSR) program	Pittsburgh Sleep Quality Index (PSQI)	T0 = Baseline • T1 = 8 weeks • T2 = 2 months	A significant medium-sized effect was found for MBSR participants with baseline PSQI scores ≥ 10, *F*_(2, 28)_ = 3.13, *p* = 0.04. These findings indicated that better sleep quality for older adults with higher levels of sleep disorders may be associated with participation in MBSR.	EG T0 = 6.22 (3.73) T1 = 5.23 (3.36) T2 = 5.44 (3.32) CG T0 = 5.29 (3.58) T1 = 5.19 (3.66) T2 = 5.10 (3.60) *F* = 3.68, *p* = 0.03. Effect size = 0.02
China	Zhang et al. (36)	Older adults with sleep disorders	CG = 30 • EG = 30	Wait-list control group (WLC)	CG = 77.63 (3.01) • EG = 78.57 (2.94)	Eight-week MBSR group with 2-h classes and a 0.5-day retreat	Pittsburgh Sleep Quality Index (PSQI)	T0 = Baseline • T1 = 8 weeks	MBSR group showed a decrease in the overall PSQI score (Cohen's *D* = 1.12), while the control group did not (Cohen's *D* = −0.06). This showed that the MBSR program could be a beneficial treatment for chronic insomnia in adults over 75.	EG T0 = 11.50 (3.28) T1 = 8.17 (2.61) Cohens's *d* = 1.12 CG T0 = 1.27 (3.62) T1 = 11.47 (3.58) COHENSD = −0.06. *F* = 8.121 *p* = 0.006
USA	Black et al. (37)	Older adults with sleep disorders	CG = 24 • EG = 25	Sleep hygiene education (SHE) intervention of 6 weeks (2 h per week)	66.3 (3)	Standardized mindfulness practices intervention of 6 weeks (2 h per week)	Pittsburgh Sleep Quality Index (PSQI)	T0 = Baseline • T1 = 6 weeks	The MAP group showed significant improvement compared to the EHS group in terms of the secondary health outcomes of insomnia symptoms, depression symptoms, fatigue interference, and fatigue severity (*p* < 0.05 for all).	MAP, media T0 = 10.2 (1.7) T1 =7.4 (1.9) With SHE intervention, T0 = 10.2 (1.8), T1 = 9.1 (2.0). Mean difference between the groups of 1.8 (IC 95 %, 0.6–2.9) with an effect size of 0.89.
USA	Irwin et al. (38)	Older adults with chronic and primary insomnia	CG = 48 • TCC/25SS • EG = 50	Tai chi or sleep seminar education control (SS) for 2-h group sessions weekly over 4 months with follow-up at seven and 16 months.	66.3 (7.4)	CBT, traditional therapies, or SS for 2-h group sessions weekly over 4 months with follow-up at seven and 16 months.	Insomnia diagnosis according to DSM-IV-TR criteria using a structured interview and checklist, performed by the study psychiatrist (MRI)/Pittsburgh Sleep Quality Index (PSQI)/Athens Insomnia Scale, AIS	T0 = baseline T1 = 2 months • T2 = 3 months • T3 = 4 months • T4 = 7 months T5 =16 months	CBT performed better than traditional therapies and SS in terms of the remission of clinical insomnia as determined by a physician (*P* < 0.01). CBT also showed greater and more sustained improvement in sleep quality and sleep parameters than TCC and SS (all *P* < 0.01).	CBT resulted in a nearly two-fold greater rate of remission than TCC and SS (χ^2^ = 9.34, *P* < 0.01) T0 = 10.4 (2.9) T1 = 7.1 (2.8) T2 = 6.6 (2.8) T3= 6.4 (2.6) T4 = 5.5 (2.5) T5 = 5.6 (3.0). CBT resulted in greater improvements in global sleep quality than TCC (t531.3 = 2.64; *P* < 0.01; estimated *d* = 0.27) and SS (t522.7 = 2.70; *P* < 0.01; estimated *d* = 0.44) from baseline to 16 months
China	Wong et al. (39)	Older adults with sleep disorders	CG = 46 • EG = 48	Sleep hygiene education and exercise program (SHEEP)	72 (5.3)	Mindfulness-based therapy for insomnia (MBT) I8 weekly 2-h group sessions, plus daily practice.	Macroarchitecture (N2, N3, and REM), and microarchitecture (sleep fragmentation, slow wave activity, spectral band power) measured by ambulatory polysomnography (PSG).	T0 = Baseline • T1 = 8 weeks	The MBTI (Myers-Briggs Type Indicator) test and sleep hygiene education had different effects on sleep macro- and microarchitecture, suggesting that the underlying mechanisms of mindfulness training to improve sleep quality may differ from traditional interventions. In short, the way mindfulness affects sleep quality may be different from other traditional techniques used to improve sleep.	SOL 2.05 *P* < 0.001 SOL pre 22.46 (17.63) post 20.40 (20.52)

IG, Intervention group; CG, Control group; T, Type; D, Duration; F, Frequency; SD; Standard deviation; CBCT, Computer-based cognitive training; TCT, Traditional cognitive training; CT, Cognitive training; CSDD, Cornell Scale for Depression in Dementia; GDS, Geriatric Depression Scale; CDR, Clinical dementia rating; IADL, Instrumental Activity of Daily Living; ICS, individual cognitive stimulation; MMSE, Mini-mental state examination; MoCA, Montreal Cognitive Assessment; TAU, Treatment-as-usual group; CST, Cognitive stimulation therapy; BDI, Beck Depression Inventory; NE, Neuroeducation; MADRS, Montgomery-Åsberg Depression Rating Scale; CBT, Cognitive behavioral therapy.

### 3.4 Study results

All ten articles (30–39) evaluating the effects of mindfulness-based cognitive therapy on older adults with sleep disorders) obtained statistically significant results after the intervention. This was evidenced by changes in the Insomnia Severity Index (ISI), Pittsburgh Sleep Quality Index (PSQI), and SOL (Sleep Onset Latency), evaluated by polysomnography (PSG).

After 8 weeks of treatment, Camino et al. (30) achieved a significant decrease in ISI values [*F*_(10.41)_; *p* = 0.002] in older adults with insomnia. With the same intervention time, Perini et al. (33) found that the time interaction × group [*F*_(125.1)_ = 6.89, *p* = 0.010] showed a significantly greater reduction in insomnia severity than the control group; while Wong et al. (32) demonstrated significant changes in ISI, *p* = 0.023, but with a small effect size (−0.360 CI 95: −0.675, −0.046). On the other hand, Shaif et al. (31) highlighted that the discrepancy in sleep onset latency, measured by polysomnography and actigraphy, decreased significantly (SOL Dif = 18.24 *p* = 0.036). Similarly, Wong et al. (39) reported SOL differences: pre 22.46 (17.63) and post 20.40 (20.52) *p* < 0.001 (10).

At 8 weeks post-intervention, Zhang et al. (36) identified a decrease in the global score of the PSQI (Cohen's D = 1.12). Gallegos et al. (35) found a significant medium-sized effect for a change in PSQI, *F* = 3.13, *p* = 0.04; while in the research developed by Lee et al. (34), changes were evidenced (PSQI: 8.3–6.7, *p* < 0.001) that were maintained at 2 months and 4 months post-intervention. Irwin et al. (38) employed a 4-month intervention that showed changes in PSQI values (χ^2^ = 9.34, dif means 2.64; *P* < 0.01; estimated *d* = 0.27), while Black et al. (37) demonstrated mean between-group differences for the same variable (1.8 95% CI, 0.6–2.9), with an effect size of 0.89 after only 6 weeks of treatment (Table 1).

### 3.5 Meta-analysis

**Figure 2:**
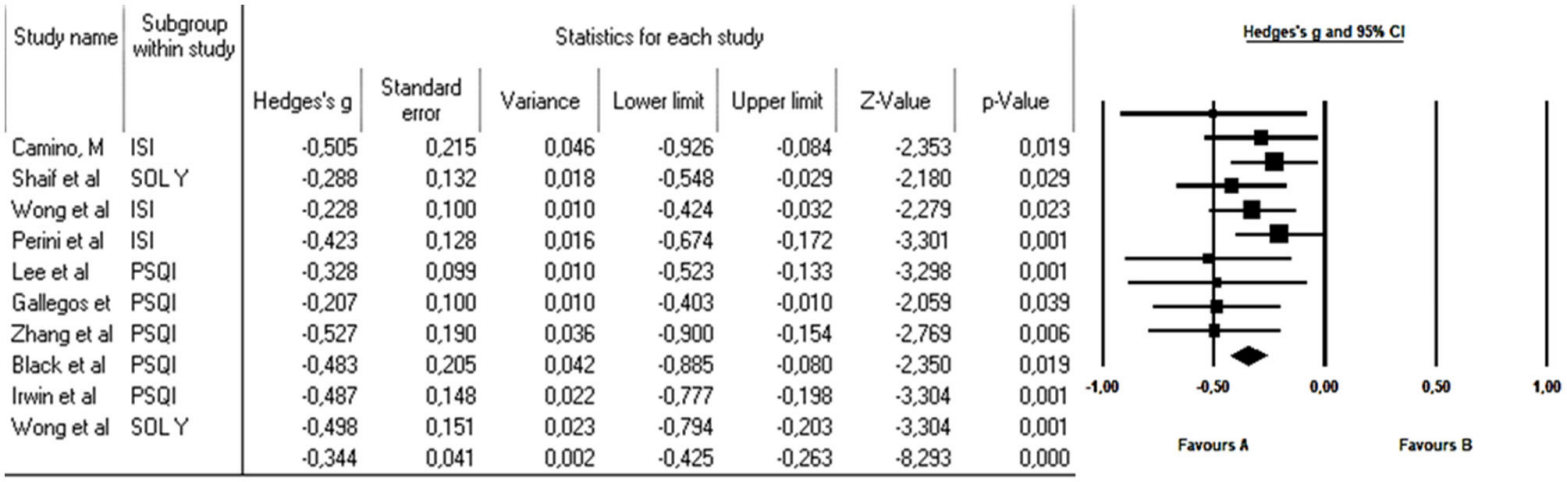
Forest plot effect of mindfulness-based cognitive therapy on older adults with sleep disorders. The black box represents the point estimate for each respective study, while the box size represents the population size and the horizontal line is the 95% CI. The diamond-shaped figure represents the estimated point of the mean difference.

All 10 articles could be integrated into the meta-analysis to synthesize the findings. The heterogeneity analysis showed that the value of *Q* was 7.740 with nine degrees of freedom. The I-squared statistic, which quantifies the percentage of variability in observed effects attributed to real effects rather than sampling error, was set at 0%. Furthermore, we calculated Tau-squared and Tau, which provided insights into the variance and standard deviation of true effect sizes in d units, respectively. Both Tau-squared and Tau were computed as 0.000, suggesting that all studies shared a common effect size without any dispersion of true effects. Lastly, with regard to the prediction interval, it was not reported because our analysis estimated Tau-squared as zero, reinforcing the notion that all studies exhibited consistent effect sizes without any variability in true effects. This comprehensive analysis, incorporating these statistical measures, provides valuable insights into the results and the homogeneity observed in effects among the selected studies. Since the heterogeneity indexes I-squared, tau-squared, and tau are minimal, the fixed-effect model was used for the analysis. The effect size index used was the standardized difference between the means (g), −0.344, with a 95% confidence interval of −0.425 to −0.263 (Figure 2).

### 3.6 Subgroup analysis

Subgroup analysis was conducted using the three sleep-disturbance measurement tools, the outcomes revealed notable statistical significance, underpinned by moderate and inversely negative Hedge's g effect sizes; Specifically, the results indicated an effect size of −0.326 for the ISI, −0.343 for the PSQI, and −0.28 for the SOL, showcasing the substantial impact of these respective measurement tools on the observed outcomes. Subgroup analyses based on the assessment tool demonstrated consistent effect sizes in all cases. This consistency in our results suggests that the choice of the assessment tool had a minimal impact on the observed treatment effects.

**Figure 3:**
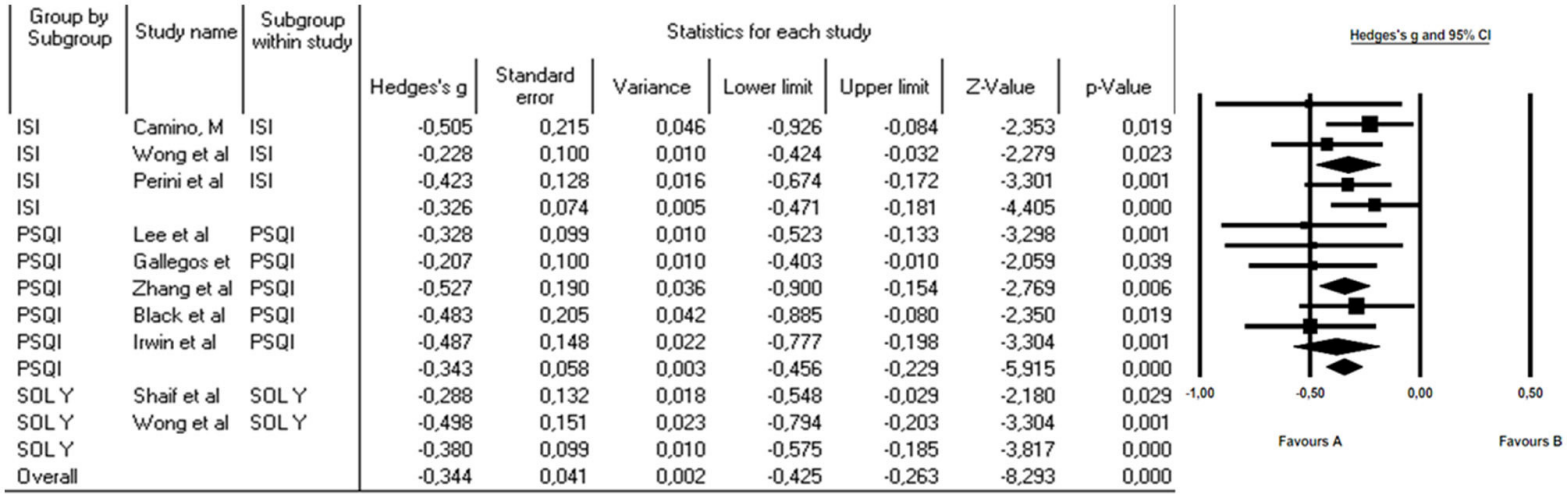
Subgroup analysis to assess the effect of MBCT on the Insomnia Severity Index (ISI), Pittsburgh Sleep Quality Index (PSQI), and SOL (Sleep Onset Latency).

Independent *Q*-tests were conducted for each of the subgroups. In the ISI Subgroup, no evidence of significant heterogeneity was found, as the *Q*-value was 5.2 with 3 degrees of freedom (df) and a *p*-value of 0.16. Similarly, in the PSQI Subgroup, the *Q*-value was 4.5 with 2 df and a *p*-value of 0.11, also indicating the absence of significant heterogeneity. In contrast, the SOL Subgroup exhibited significant heterogeneity, with a *Q*-value of 10.8 and 4 df, along with a *p*-value of 0.03. These results suggest that the variability in treatment effects is more pronounced in the group of patients assessed with the SOL instrument compared to the other two groups, where heterogeneity is insignificant (Figure 3).

### 3.7 Publication bias

**Figure 4:**
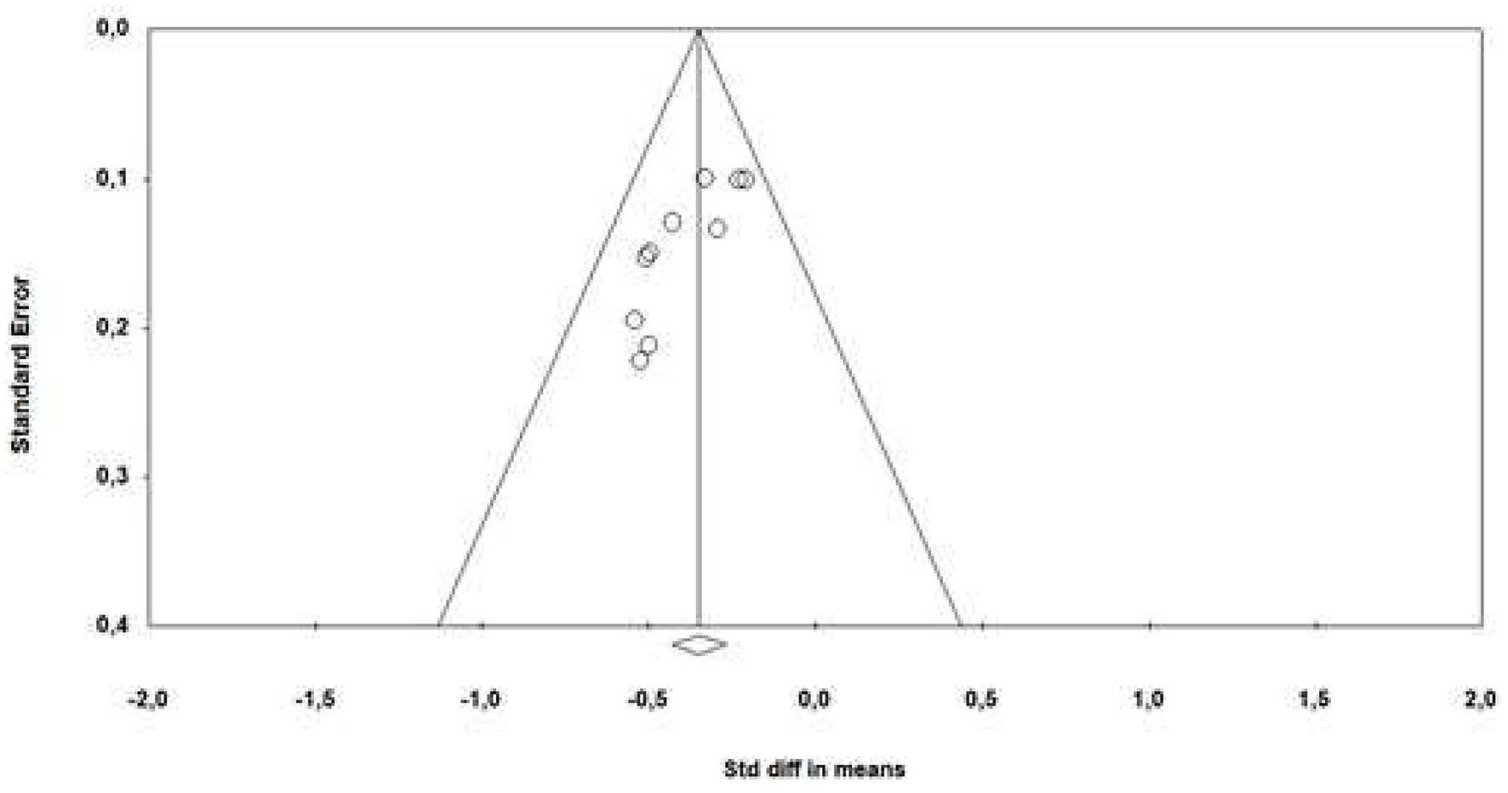
Funnel plot of standard error by standard differences in medias.

The publication bias analysis was performed using a funnel plot (Figure 4) that included all the articles in the meta-analysis. An expected publication bias was revealed as various articles showed different mean difference results. However, when a subgroup analysis was performed based on the assessment instrument used, heterogeneity was observed to decreased, thus producing a more symmetrical distribution of results.

### 3.8 Quality of evidence

For each review outcome, quality of evidence was assessed (Table 2) using the Grading of Recommendations Assessment Development and Evaluation (GRADE) framework (40–42). An a priori ranking of “high” was assigned given that all studies included were randomized controlled trials. Evidence quality was downgraded a level if one single study presented a high risk-of-bias (failure to achieve “low risk” in two or more criteria included in the risk-of-bias assessment presented in Table 3, or the majority of studies suffered from the same risk of bias. Evidence quality was also downgraded if inconsistent findings imprecision, indirectness, and publication bias were reported. The quality of evidence was rated as high, moderate, low or very low.

**Table 2:** Quality of evidence.

**Outcomes**	**Number of participants (studies)**	**Quality of evidence (GRADE)**
Insomnia severity	221 (3 studies)	⊕⊕⊕○ Moderate^b^
Sleep quality	790 (5 studies)	⊕⊕⊕⊕ High
Sleep onset latency	207 (2 studies)	⊕⊕⊕○ Moderate^a, c^

a High risk of bias, downgraded with one level.

b Inconsistent findings, downgraded with one level.

c Imprecision due to the small number of participants, downgraded with one level.

**Table 3:** Methodological quality of the articles included.

**References**	**1**	**2**	**3**	**4**	**5**	**6**	**7**	**8**	**9**	**10**	**11**	**Total**
Camino et al. (30)	Y	Y	Y	Y	Y	N	N	Y	Y	Y	Y	8
Shaif et al. (31)	Y	Y	Y	Y	Y	Y	N	Y	Y	Y	Y	9
Wong et al. (32)	Y	Y	Y	Y	N	N	N	N	Y	Y	Y	6
Perini et al. (33)	Y	Y	N	Y	N	N	Y	Y	Y	Y	Y	7
Lee et al. (34)	Y	Y	Y	Y	N	N	Y	Y	Y	Y	Y	8
Gallegos et al. (35)	Y	Y	N	Y	N	N	N	Y	Y	Y	Y	6
Zhang et al. (36)	Y	Y	Y	Y	Y	Y	N	Y	Y	Y	Y	9
Black et al. (37)	Y	Y	Y	Y	N	N	N	Y	Y	Y	Y	7
Irwin et al. (38)	Y	Y	Y	Y	Y	Y	N	Y	Y	Y	Y	9
Wong et al. (39)	Y	Y	Y	Y	N	N	N	Y	Y	Y	Y	7

Items: 1 = eligibility criteria; 2 = random allocation; 3 = concealed allocation; 4 = baseline comparability; 5 = blind subjects; 6 = blind therapists; 7 = blind assessors; 8 = adequate follow-up; 9 = intention-to-treat analysis; 10 = between-group comparisons; 11 = point estimates and variability. Y, Yes; N, No.

## 4 Discussion

This systematic review and meta-analysis aimed to analyze the effectiveness of a mindfulness-based program on sleep quality in healthy non-institutionalized older people. The review included a total of 10 articles that met the eligibility criteria and utilized mindfulness as the main treatment for improving sleep quality (30–39). The results indicated that a mindfulness program improved sleep quality in older adults with sleep disorders.

Poor sleep quality is a common problem associated with several adverse effects on the physical, mental, and social wellbeing of older adults (43). Moreover, sleep problems have been shown to increase considerably with aging (41). In this systematic review, although most studies used the Pittsburgh Sleep Quality Index as the instrument for assessing sleep quality (30, 33–38), others used the Insomnia Severity Index (30–33), and two used polysomnography (31, 39). Therefore, a meta-analysis could be performed in which a subgroup analysis was performed. This analysis was carried out using the three tools for measuring sleep disturbances presented in the selected articles and the findings showed notable statistical significance supported by moderate and inversely negative Hedge's g effect sizes, being consistent in all cases.

Various pharmacological interventions have been undertaken to improve sleep problems; however, in addition to the well-known risks of sleep medications, no lasting improvements in sleep outcomes have been demonstrated after discontinuation (8). The results of this review suggest that non-pharmacological intervention approaches to address sleep quality problems are more effective, safer, and preferable compared to sleep medications. Some therapies, such as cognitive behavioral therapy, are not widely available since they must be administered by a trained and licensed specialist (3). In addition, healthcare providers have no established practice guidelines or standards for the combined use of individual therapies, so they may opt for sleep medications (44). Another non-pharmacological strategy carried out in all the studies selected for this systematic review and meta-analysis to address sleep quality problems was the mindfulness-based intervention, which has demonstrated beneficial effects in older adults (45, 46). First used as an intervention approach for treating chronic stress, anxiety, depression, and even pain, mindfulness has also proven effective in preventing falls (47), reducing systemic inflammation (48), and reversing metabolic disease (49) in older people. A qualitative study (50) found that older adults lose work-related activity and stress after retirement, so mindfulness can become an opportunity to increase physical activity, decrease inflammatory factors, reduce anxiety, and, therefore, improve sleep quality. A previous meta-analysis has also shown that in cancer patients, although mindfulness had a lesser effect than aerobic exercise, both interventions significantly improved sleep problems (51). Similarly, the current study adds to the highly comprehensive evidence indicating the effect of MBI on sleep quality.

Likewise, the analysis showed that mindfulness for 16 months (38) or, alternatively, 2 months (32, 35) of intervention, had similar effects on sleep that were maintained even at 2 and 4 months after the intervention (34). Thus, its effectiveness was not only short-term but also long-term. It must also be highlighted that three of the articles selected (31, 36, 38) for this review had excellent quality and the remaining seven (30, 32–35, 37, 39) were rated as good, so the relatively high quality of the included RCTs makes our conclusions comparatively reliable. Together with the high rate of adherence in the participation of these studies, it can be argued that the treatment and evaluation protocol is feasible in this population. Similarly, these types of interventions provide feedback to patients, motivating them to fully accept their new experiences. Therefore, these interventions must be incorporated due to the better quality of life and acceptance they bring to people at an age when a series of changes occur that negatively affect their health. However, therapists have yet to incorporate these approaches consistently into their recommendations, despite the evidence of their effectiveness and safety.

Meanwhile, several sleep improvement interventions focused on physical exercise therapy have been carried out but with mixed results. Some researchers have suggested that mild to moderate exercise may improve self-perceived sleep quality and alleviate symptoms of sleep deprivation in older people after an intervention (52–56). In addition, the main advantages of exercise programs include their low cost, accessibility, and lack of adverse effects (57). However, only limited evidence is available of the lasting benefits of exercise for improving long-term sleep problems (3, 58, 59). Similarly, light exposure therapy has been conducted for sleep improvement, but this has produced insufficient evidence of consistent improvements in long-term sleep quality, even compared to a control group that followed no treatment (3, 59). Conversely, mindfulness interventions, computer-based cognitive training, or other multidimensional approaches that integrate certain underlying causes of poor sleep quality, primarily chronic stress have shown promise for improving sleep later in life (8, 44, 58). In addition, these approaches avoid potential adverse effects and safety issues, do not need to be delivered by highly trained professionals with specific certifications, and are more accessible. Some even use low-cost online sessions as a delivery method (37, 60). Mindfulness has also produced results in a shorter period and it can be practiced anytime and anywhere (61). Therefore, for several reasons, multidimensional non-pharmacological intervention approaches have several distinct advantages that could make them ideal for older adults experiencing poor sleep quality.

The current study has certain limitations. First, four databases were used to search for studies published in English, which may limit the generalizability of the results to some extent. Ongoing database updates and replenishment will be considered in the future. Second, only a limited number of studies and a relatively small overall sample size provided the physiological measures of sleep duration. Although significant effects on subjective sleep outcomes were observed, no improved sleep quality could be detected through physiological parameters. More research on subjective and physiological sleep is needed in future studies. Third, most studies were conducted in Asia and the Americas, so a geographic bias is identifiable, making it impossible to generalize the results to populations in other regions. Finally, gender differences were not considered in the meta-analysis of this review because the selected studies only included older adult participants with no gender distinctions.

## 5 Conclusion

Following this systematic review with meta-analysis of published data to assess the effects of mindfulness on sleep quality in older people, it is suggested that mindfulness could be introduced to remedy poor sleep quality in older people in both the short and long term. Therefore, it could be a viable treatment option for insomnia or other problems related to poor sleep quality in this population. In addition, the available evidence regarding this therapy remains limited and the methodological quality of the evidence must be more rigorous. Considering that mindfulness programs can be easily offered in many communities, outreach efforts would not be a barrier in this case. Therefore, older adults would generally have immediate access to these programs, which are offered at low cost. It must be emphasized that further research with more structured quality randomized controlled trials, as well as standardized and comparable protocols, is needed to determine the relative position of meditation-based therapies among treatment options. More studies are also needed to determine whether mindfulness is a better, worse, or equivalent strategy to other types of meditation training.

## Author contributions

AG-M and AM-G: conceptualization. AA-A and YC-C: methodology. YR-C: formal analysis. AA-A and AM-G: writing—original draft preparation. AG-M and YC-C: writing—review and editing. YR-C and AA-A: supervision. All authors contributed to the article and approved the submitted version.
